# Processing of Acoustic Information in Lexical Tone Production and Perception by Pediatric Cochlear Implant Recipients

**DOI:** 10.3389/fnins.2019.00639

**Published:** 2019-06-20

**Authors:** Mickael L. D. Deroche, Hui-Ping Lu, Yung-Song Lin, Monita Chatterjee, Shu-Chen Peng

**Affiliations:** ^1^Department of Psychology, Concordia University, Montreal, QC, Canada; ^2^Chi-Mei Medical Center, Tainan, Taiwan; ^3^Taipei Medical University, Taipei, Taiwan; ^4^Boys Town National Research Hospital, Omaha, NE, United States; ^5^United States Food and Drug Administration, Silver Spring, MD, United States

**Keywords:** lexical tone, cochlear implant, cue trading, speech production, children

## Abstract

**Purpose:** This study examined the utilization of multiple types of acoustic information in lexical tone production and perception by pediatric cochlear implant (CI) recipients who are native speakers of Mandarin Chinese.

**Methods:** Lexical tones were recorded from CI recipients and their peers with normal hearing (NH). Each participant was asked to produce a disyllabic word, *yan jing*, with which the first syllable was pronounced as Tone 3 (a low dipping tone) while the second syllable was pronounced as Tone 1 (a high level tone, meaning “eyes”) or as Tone 4 (a high falling tone, meaning “eyeglasses”). In addition, a parametric manipulation in fundamental frequency (F0) and duration of Tones 1 and 4 used in a lexical tone recognition task in Peng et al. (2017) was adopted to evaluate the perceptual reliance on each dimension.

**Results:** Mixed-effect analyses of duration, intensity, and F0 cues revealed that NH children focused exclusively on marking distinct F0 contours, while CI participants shortened Tone 4 or prolonged Tone 1 to enhance their contrast. In line with these production strategies, NH children relied primarily on F0 cues to identify the two tones, whereas CI children showed greater reliance on duration cues. Moreover, CI participants who placed greater perceptual weight on duration cues also tended to exhibit smaller changes in their F0 production.

**Conclusion:** Pediatric CI recipients appear to contrast the secondary acoustic dimension (duration) in addition to F0 contours for both lexical tone production and perception. These findings suggest that perception and production strategies of lexical tones are well coupled in this pediatric CI population.

## Introduction

Cochlear implants (CIs) are medical devices that are surgically inserted in the cochlea of patients with severe-to-profound sensorineural hearing loss to provide auditory sensation by electrically stimulating the auditory nerve. Even though CI devices help to improve speech perception by patients, the device technology has its limitations. One constraint is that CI devices are equipped with speech-coding strategies that are temporal-envelope based (Shannon, 1983; Zeng, 2002), and their audio signals are delivered with poor spectral resolution. With this limitation, speech and other sound information involving complex harmonic pitch (fundamental frequency or F0) –critical for functions such as the perception of prosody (i.e., speech intonation and lexical tones), talker-sex, melody identification, and speech perception in noise – is poorly processed by CI patients (Qin and Oxenham, 2003; Wu and Yang, 2003; Fu et al., 2004; Kong et al., 2005; Galvin et al., 2007; Gfeller et al., 2007; Chatterjee and Peng, 2008; Cullington and Zeng, 2008; Luo et al., 2008; Peng et al., 2008, 2009; Zhu et al., 2011; Wang et al., 2013; Zhou et al., 2013; Chatterjee et al., 2015; Liu et al., 2019). For native speakers of a lexical tone language such as Mandarin or Cantonese, the aforementioned limitation hinders CI users’ ability to identify contrasts in lexical tones, since F0 serves as the primary information for this task (e.g., Whalen and Xu, 1992; Liu and Samuel, 2004). This limitation is particularly challenging for pediatric CI recipients who are prelingually deaf (i.e., born deaf or become deaf before ages five or six), given that these individuals have to rely on a CI to master the lexical tone system critical for their spoken language development. The restricted access to F0 information may also affect how pediatric CI listeners utilize F0 cues along with secondary acoustic dimensions, such as duration, to identify as well as produce lexical tones.

### Lexical Tone Perception

Lexical tone *perception* has been widely studied in both adult and pediatric patients with CIs. Wang et al. (2013) examined lexical tone recognition using mono-syllabic words in CI patients who were post-lingually deaf. They found that performance was much poorer than adult listeners with normal hearing (NH), and also poorer than adult individuals with severe hearing loss. They observed a negative correlation between performance and audiometric thresholds of adult listeners who are hearing impaired, particularly at 250 Hz, highlighting the critical importance of low frequencies for this task. Wang et al. (2011, 2012) found performance in this task to be correlated with complex pitch discrimination and musical instrument identification. However, adult listeners with a long CI device experience are known to alter listening strategies to perform auditory tasks. As their device does not allow a fine representation of F0 contours, post-lingually deaf adult CI users have been shown to develop alternative strategies for lexical tone recognition based on secondary (or possibly tertiary) cues. This phenomenon is referred to as *cue-trading* and has been shown in many speech perception tasks when the primary cue for the task is degraded. For instance, Peng et al. (2009, 2012) examined English-speaking CI users to distinguish questions from statements based on their contrasts in speech intonation. As the primary cue for speech intonation (F0 contour) was poorly transmitted by their devices, CI users showed greater reliance on secondary cues (intensity and duration patterns) to perform this task. Cue-trading is also observable in listeners with NH or with CI in the laboratory setting, by manipulating the type and quality of acoustic information in phonetic identification (e.g., Winn et al., 2012, 2013).

While cue-trading has been demonstrated in adult listeners, the phenomenon has been relatively under-studied in children. The literature suggests that children and adults use different sets of perceptual weights for speech recognition (Nittrouer, 1996; Hazan and Barrett, 2000). Among pediatric CI recipients who are prelingually deaf, performance in lexical tone recognition has been reported as highly variable (e.g., Ciocca et al., 2002; Peng et al., 2004; Zhou et al., 2013; Chen et al., 2014). There is, however, a consistent trend: those who perform better in lexical tone recognition tend to have longer experience with their device. This trend suggests that while cue-trading in electric hearing takes time to learn, children eventually adapt and develop novel strategies in their language. On the other hand, Chen et al. (2014) reported that while maternal education level (an indicator of socio-economic status) plays a positive role for speech recognition in children with CIs, it does not predict their lexical tone identification performance. This outcome points toward limitations inherent to the device that are not easily overcome by the development of alternative strategies or environmental factors.

### Lexical Tone Production

Lexical tone *production* by pediatric CI recipients has also been investigated in several studies. Similar to findings with lexical tone recognition, considerable individual variability was observed within each study. In addition, findings among studies exhibited discrepancies, potentially related to the different protocols and methodologies adopted among those studies. Broadly speaking, two approaches have been followed. Some studies (Peng et al., 2004; Xu et al., 2004; Han et al., 2007) asked experienced listeners (typically speech pathologists or NH adult listeners who are familiar with the speech of hard of hearing) to rate how they would perceive the accuracy of the lexical tones produced by the children. Accuracy was reported as between 30 and 70% correct for the majority of children with CI, being considerably lower than the accuracy of their NH peers. Tones 1 and 4 were generally better produced than Tones 2 and 3, a pattern consistent with the developmental trend among children with NH in their acquired mastery with lexical tones in Mandarin Chinese (Li and Thompson, 1977). However, those studies warned that it is sometimes difficult for judges to make reliable assessment about the quality of lexical tone productions that are irregular over time (across repetitions). To circumvent this issue, another approach was followed in which the recordings were either analyzed acoustically and some indices were derived to reflect the production quality (e.g., Barry and Blamey, 2004) or automatically categorized by a neural network based on F0 contours (Zhou and Xu, 2008; Xu et al., 2011; Zhou et al., 2013). This second approach thus permitted a relatively objective assessment of production quality (i.e., free from human biases). A large overlap between tonal ellipses, i.e., a lack of tonal differentiation, was reported for CI children (Barry and Blamey, 2004). Further, the outcomes of the neural network were largely consistent with NH listeners’ ratings, i.e., they confirmed substantial deficits in lexical tone production by prelingually deafened children with CI that worsened as age at implantation advanced (Zhou et al., 2013).

### A Link Between Perception and Production?

Outside of the lexical tone literature, it has been known for a while that perception and production are tightly linked (Bradlow et al., 1997; Houde and Jordan, 1998), including for F0 control (Elman, 1981; Larson et al., 2000). Naturally, this has led the aforementioned studies to focus largely (for human judges) or solely (for the acoustic analyses of Barry and Blamey (2004), or the neural network adapted from Zhou and Xu (2008) on the quality of the F0 contours produced. The rationale was that children with CIs would not be able to produce tones correctly unless they were able beforehand to perceive F0 sufficiently well to learn to recognize the particular F0 inflections of a given tone and eventually fine-tune their speech motor commands. To some degree, this rationale is supported by correlations between perception and production performance (Peng et al., 2004; Xu et al., 2011; Zhou et al., 2013). However, this rationale suffers from a serious limitation: considering the cue-trading phenomenon established in perception, information other than F0 must be examined. One might easily imagine that CI recipients deemphasize F0 contours and emphasize differences in intensity or duration while producing lexical tones, but such cue-trading phenomenon in production remains to be documented. This is important because regardless of the fact that all CI recipients suffer from some loss in functional spectral resolution, a fraction of these children exhibit little deficit in lexical tone production (Han et al., 2007; Zhou and Xu, 2008). Without explicit knowledge of the type of acoustic information being used for perception and those emphasized in production, we might not appreciate the roots of individual variability. If the reliance on specific acoustic dimensions in tone identification were reflected in production *by the same individuals*, this would suggest mechanistic links between the development of perception and production of lexical tones that are driven by the characteristics of the acoustic input.

### Goals of the Study

The purpose of this study was (1) to examine the aspects of vocal production that Mandarin Chinese speaking pediatric CI recipients emphasize or deemphasize to convey lexical tones, (2) to compare pediatric CI recipients’ lexical tone production to that of NH peers, and (3) to compare lexical tone production and perception in the same participants. The perception task followed the design of a preliminary study (Peng et al., 2017) that focused on a single, unambiguous, contrast: Tone 1 vs. Tone 4. In running speech, Tones 2 and 3 can sometimes bear some similarity and are known to be mastered relatively later throughout development (Li and Thompson, 1977). For children as young as 6 years of age, we wished to avoid any abnormal production that would solely be the result of those tones still being learnt about. Thus, the study also focused on the production of Tones 1 and 4 exclusively, which were more likely to reflect intentional and consolidated speech motor commands.

We hypothesized that in lexical tone production, NH children would contrast the two lexical tones based on F0 contours exclusively. In contrast, pediatric CI recipients would express differences in duration or intensity patterns, while not modulating their vocal chords’ vibrations well enough to contrast the high-level F0 contour of Tone 1 vs. the falling contour of Tone 4. Finally, we suspected that the perceptual weights derived for an isolated word, controlled in laboratory settings, may not necessarily generalize to perceptual strategies recruited in running speech (ecological situations), and therefore, they may not have had time to influence the speech motor commands. Thus, we expected to observe a perception-production coupling more easily in children who had more extensive experience with their device.

## Materials and Methods

### Participants

Participants in this study were comprised of 40 pediatric CI recipients who all became deaf prelingually (deafness before 2.3 years of age), ranging from 6.4 to 17.2 years of age. For the most part (37 out of 40), they were implanted early between 1.1 and 4.5 years of age; only three were implanted at 5.6, 7.3, and 12.2 years of age. Consequently, the median of age at implantation was 2.5 years. These children had used their CI devices from 1.2 to 15.2 years. Note that there was no correlation between chronological age and age at implantation [*r*^2^= 0.03, *p* = 0.295], or chronological and age at profound hearing loss [*r*^2^= 0.02, *p* = 0.427], but a significant correlation between chronological age and years of CI use [*r*^2^= 0.72, *p* < 0.001]. In addition, 35 NH children (bilateral thresholds <20 dB HL at octave frequencies between 250 and 8000 Hz) were recruited. There was no significant difference in age at testing between the CI and NH groups [*t*(73) = -0.4, *p* = 0.700]. The demographics of these two participant groups are reported in Table 1. All participants and their parents provided written informed consent, which was reviewed and approved by the Institutional Review Board at the Chi-Mei Medical Center.

**Table 1:** Demographics of the two populations of children, who had normal hearing or were wearing a cochlear implant.

	Chronological age mean (std.) [min–max]	Age at implantation mean (std.) [min–max]	Duration of CI use mean (std.) [min–max]
NH children (*n* = 35)	10.6 (2.8) [6.8–16.5]		
CI children (*n* = 40)	10.9 (3.4) [6.4–17.2]	2.9 (1.9) [1.1–12.2]	7.9 (3.6) [1.2–15.2]

*Numbers are given in years. All CI children had lost their hearing before age 2.3. Note that the distribution of age at implantation was skewed: only three children were implanted at 5.6, 7.3, and 12.2 years of age while the remaining 37 were implanted before 4.5 years of age.*

Among the CI participants, seven were implanted bilaterally, 15 were implanted unilaterally (eight on the left side, seven on the right), wearing no hearing aid on their contralateral ear. The remaining 18 were bimodal users, who wore a hearing aid on the contralateral ear (13 implanted on the left side, and 5 on the right). However, CI participants were tested (in the perception task) and recorded (in the production task) with a single CI, being the device implanted first. This CI was turned on, while using the clinically assigned settings, and any other implant or hearing aid on the contralateral ear was removed. Thirty-five participants were users with a Cochlear Nucleus device (N24, CI422, CI512, Nucleus Freedom, all using the ACE coding strategy). Four were users of a Med-El device (Pulsar, Concerto, Sonata, using the Opus2 coding strategy). One remaining participant was a user of the Advanced Bionics’ HiRes90k device, using the Fidelity 120 coding strategy. All participants with hearing aids had audiometric thresholds exceeding 90 dB HL at the time of testing, but some of them may have been exposed to acoustic hearing pre-implantation. For example, one of the participants, implanted at age 12, had high thresholds (∼80 dB HL) until he suffered sudden profound hearing loss and subsequently received a CI. Although perception and production measures were all made with only the CI in place, thus excluding any effects of acoustic hearing at the time of testing, the presence of hearing during development may be expected to influence perceptual cue-weighting as well as production patterns. Therefore, we included an analysis based on the presence or absence of residual hearing in our participants.

### Production Task

All participants were asked to produce the Chinese disyllabic word “yan-jing,” with the 2nd syllable pronounced with Tone 1 (a high level tone) and Tone 4 (a high falling tone) to represent “eyes” (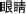) and “eyeglasses” (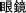), respectively. The first syllable is always pronounced with Tone 3 (a dipping tone). Participants were asked to produce the target words in a natural way, just as how they would say it in everyday life. Three repetitions of each target word were recorded from each participant, in order to increase the number of observations and determine to what extent the extracted acoustical parameters varied from one recording to another. The recordings were performed at two clinical sites, the Chi-Mei Medical Center in Tainan and the Chang Kung Memorial Hospital in Taoyuan. The experimental sessions were set up at both sites in the following, comparable fashion: Signals were recorded at a 44.1 kHz sampling rate with a 16-bit resolution, with a minidisc recorder (Sony MZ-RH1) through a stereo microphone (Audio-Technica AT9440) placed approximately 10 cm from the speaker, in a sound-treated booth. The recordings were transferred from the mini disc to a laptop through the Sonic Stage (Digital Music Manager Version 3.4) software program and saved as .wav files for further editing. With the Adobe Audition 3.0 software program, each of the signals was cut with 400-ms of silence before the onset and after the offset.

### Perception Task

The perception task followed the same methods identical to that in Peng et al. (2017). A continuum between Tones 1 and 4 was created in the lab by orthogonally manipulating (1) the slope of the F0 contour, and (2) the duration, of the second syllable of the word “yan-jing.” The range of slopes varied from -1, -0.8, -0.6, -0.4, -0.3, -0.2, -0.1, to 0 octave. The range of durations varied from 40, 60, 80, 100, 120, and 140% of the initial duration. These manipulations were performed at a F0 height of 120 Hz (typical of male voices) or 220 Hz (typical of female voices), resulting in a total of 96 tokens per testing session. Each participant completed three or four sessions, in which all tokens were presented one after another in random order. This task followed a single-interval, two-alternative forced-choice paradigm (2AFC) in which the participant was asked to indicate whether a given stimulus meant “eyes” or “eyeglasses” by clicking on one of two response buttons shown on the computer screen. Sounds were delivered from an external soundcard (Soundmax Integrated HD Audio) connected to a laptop through loudspeakers (Audio Pro) at approximately 65 dB SPL at the child’s ears. Although the amplitude contour (which is naturally correlated with the F0 contour, at least within the NH population) conveys some information about lexical tones (Whalen and Xu, 1992), the overall intensity does not. All stimuli were therefore, presented at the same root-mean-square (RMS) level. The amplitude contour was not manipulated in this study, as the study focused on the trade-off between F0 and duration cues.

## Production Data Analysis

### Extracting Acoustic Parameters

Acoustic analyses were performed on each of the two syllables for all recorded tokens. We extracted the intensity pattern sampled every 5 ms with Praat (Boersma and Weenink, 2018), and ran a peak detection algorithm with a peak prominence of 20 dB. In 8 cases, this algorithm did not permit us to successfully locate the two peaks because the intensity pattern dropped by less than 20 dB between the two syllables. When this occurred, the peak detection algorithm was reiterated with a lower peak prominence until it successfully located two peaks. Each syllable was then trimmed on either side of the intensity peak. The choice of 20-dB cutoff permitted selection of the entire syllable, including the last phoneme /n/ in the first syllable or /η/ in the second syllable. The F0 values were also sampled every 5 ms. All recordings were first concatenated together and F0 points were extracted within a default range 75 to 600 Hz. This resulted in a dominant distribution with a few outliers that were octave jumps. To prevent those errors, the F0 distribution was then fitted with a *normal* probability density function on a logarithmic axis, essentially to reflect the vocal range of a given child. The mean of the fit was chosen as the center of the vocal range which was subsequently restricted to +/-6 semitones around. Each token was then analyzed using Praat with this narrow F0 range. Visual inspections were performed to identify any abnormality. Abnormalities occurred in four occasions for the NH population and 11 occasions for the CI population over all tokens, either because (1) the production was not sufficiently voiced, or because (2) the F0 contour exceeded the +/-6 semitones range (e.g., higher range for Tones 1 than 4). In cases (1), the voicing threshold was adjusted manually to 0.1 rather than the default 0.45 (parameter in Praat) as a way to reduce the influence of unvoiced portions (while keeping a 20-dB cutoff window). In cases (2), the F0 range was expanded up to +12 semitones and down to -9 semitones relative to the center of the vocal range. The entire F0 contour was recorded, but for the purpose of this study, the analyses focused on two descriptors: F0 median and F0 movement from the first to the last 30 ms.

**FIGURE 1:**
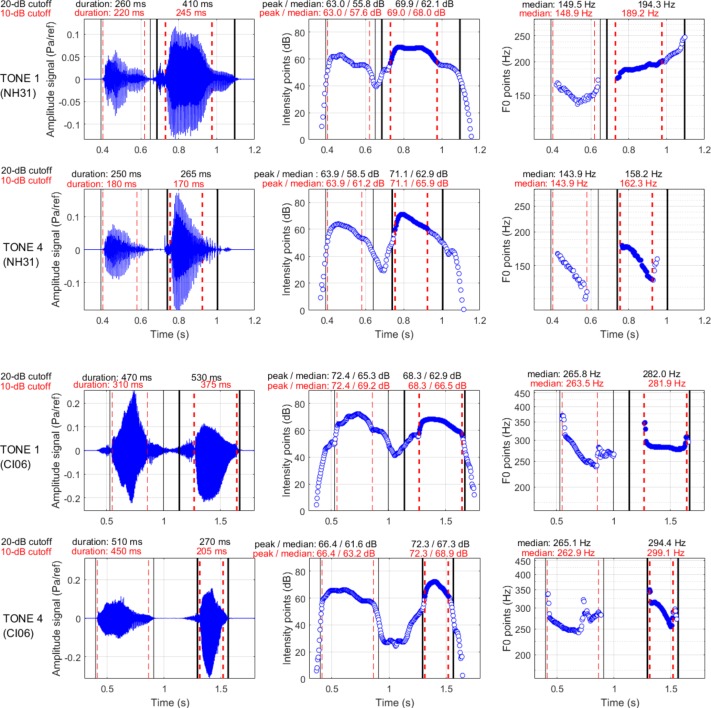
The “yan-jing” production, with the second syllable produced as Tone 1 (top) or Tone 4 (bottom) by a 12.7-year-old participant with NH (top two rows) and by a 10.9 year-old participant with a CI (bottom two rows).

An additional analysis was performed with a 10-dB cutoff, revealing qualitatively similar findings as with the 20-dB cutoff (see Appendix). Its rationale was that the final phoneme (voiced consonant) contributed in some cases to the F0 contours of Tones 1 and 4 (e.g., right panels Figure 1). Since the middle vowel was more intense than the phonemes embedding it, this procedure allowed a closer focus on the voiced part of each syllable, which provided more canonical F0 contours even though it was too conservative.

As an example, Figure 1 shows the parameters extracted from the recordings of Tones 1 and 4 produced by a male NH participant (top two rows) and by a female CI participant (bottom two rows). In each panel, the black vertical lines delimit the window selected from the 20-dB cutoff relative to the intensity peak, and the red dashed lines delimit the window selected from the 10-dB cutoff. Several traits can be observed. For the NH boy, there was little difference in duration between the two syllables; for both tones, the intensity was greater for the second than for the first syllable. For the CI girl, the syllable produced as Tone 4 was markedly short (possibly due to extended duration of the first syllable); the syllable produced as Tone 1 was markedly soft (possibly due to greater intensity of the first syllable). As shown in the right panels, the F0 pattern of the first syllable was either V-shape or falling. This pattern was quite common, and occurred whether the following syllable was Tone 1 or Tone 4. As anticipated, the boy produced the second syllable either with a higher-level/slowly rising pattern for Tone 1 or a rapidly falling pattern for Tone 4. The girl produced a falling F0 for Tone 4 but produced a largely monotonous pattern for Tone 1 that was similar to the F0 range of the first syllable.

### Statistical Analyses

The statistical analysis was performed for one acoustical parameter at a time. We used a linear mixed effects (LME) approach, where the initial model had two fixed effects: hearing status (NH vs. CI) and lexical tone contrast (Tone 1 vs. Tone 4), including an interaction term. We also considered random intercepts for each participant as well as a random slope for the effect of contrast because both of these additions significantly improved the initial model. Any other addition (random intercepts for “repetitions,” random slopes for the effect of hearing status, random slopes for the effect of sex, random slopes for the effect of chronological age, or even sex as a third fixed effect) did not improve the model and were therefore, excluded. Thus, the final model was of the form “parameter ∼ 1 + Contrast^∗^Hearing + (1+Contrast | Participant).

## Perception Data Analysis

Data from all testing sessions were pooled together and the proportion of Tone 1 responses served as the dependent variable in a logistic mixed-effect analysis. There were three fixed factors: (1) population, (2) slope of F0 variation, and (3) duration, including interaction terms. Note that the duration scale was log-transformed for centering purposes. We also included a random intercept per subject, and random slopes for the effect of F0-slope, duration, as well as F0-height. Thus, the final model was of the form “responses ∼ Population^∗^F0variation^∗^Duration + (1+F0variation+Duration+F0height | Participant).” This enabled extraction of coefficients for each subject that reflected the reliance on pitch or duration cues, which could then be correlated against the production outcomes.

## Production Results

### Duration

**FIGURE 2:**
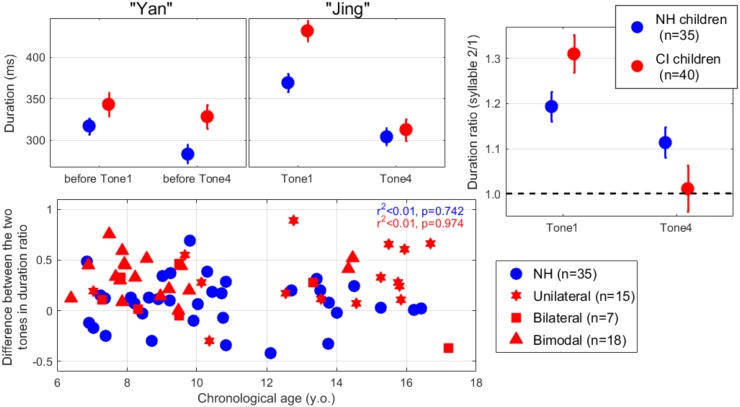
(Top left and middle) Duration of the two syllables of “yan-jing” produced as Tone 1 or Tone 4, averaged across participants in each population. (Top right) Duration ratios between two syllables indicate that children with CIs prolonged the duration of tone 1 in order to convey what is supposedly a high-flat pitch contour. Error bars show ±1 standard error of the mean. (Bottom) Difference in duration ratio between the two lexical tones, plotted across participants as a function of their chronological age. A greater positive value indicates a stronger tendency to prolong Tone 1 or shorten Tone 4. Regardless of their mode of hearing in everyday life, children with CIs were tested only a single (implanted) side.

As displayed in the top panels of Figure 2, children with NH prolonged the duration of the second syllable by about 10–20% relative to the first syllable. In contrast, participants with CIs did so for Tone 1 (by about 30%) but not for Tone 4. In other words, participants with CIs tended to contrast the duration patterns to distinguish between Tones 1 and 4. The LME was further performed on the duration ratio between the two syllables (top-right panel). This ratio permitted to discard variances associated with individual speaking characteristics, i.e., different speaking rates among participants. There was an effect of hearing status [*t*(446) = 2.2, *p* = 0.029], no effect of contrast [*t*(446) = -1.9, *p* = 0.062], and an interaction between the two [*t*(446) = -3.8, *p* < 0.001]. This interaction was the key evidence that participants with CIs utilized duration to contrast Tones 1 and 4, whereas participants with NH did not.

A question of interest was whether, there was a particular profile of pediatric CI recipients who displayed this “alternative behavior,” i.e., shortening Tone 4 or prolonging Tone 1 as a substitute for their respective F0 contours. The bottom panel shows the difference between the duration ratios of Tones 1 and 4. Here, a positive value indicates that the participant produced a longer duration for Tone 1 than for Tone 4 (still with the 2nd syllable duration being relative to that of the first syllable). This alternative behavior was shared by most of the children with CIs (with two exceptions), and was not found to be related to chronological age (*p* = 0.974). There was also no evidence that this alternative behavior was driven by age at implantation or duration of CI experience (respectively, *p* = 0.136 and *p* = 0.450, not shown).

### Intensity

**FIGURE 3:**
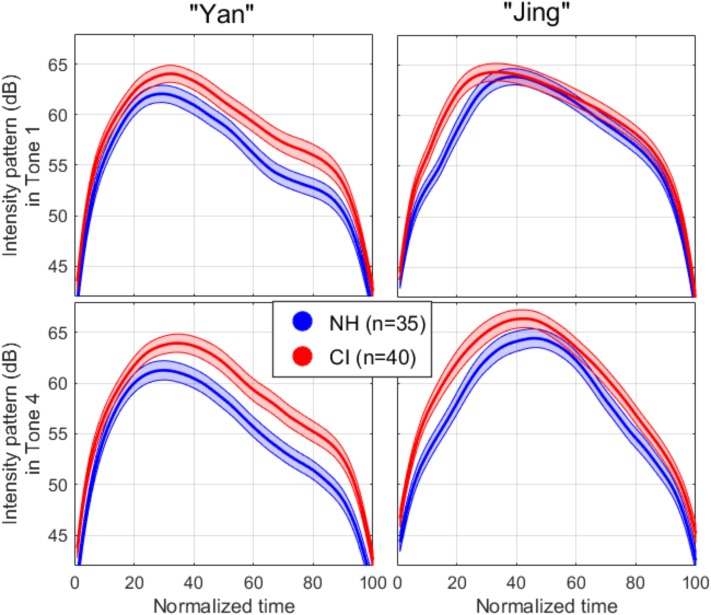
Mean (thick line) and one standard error (areas) for the intensity patterns extracted for each syllable when producing “yan-jing” with Tone 1 (top panels) vs. Tone 4 (bottom panels). The patterns were delimited in time with a 20-dB cutoff from the intensity peak, and the resulting duration was normalized from 0 to 100% of the total duration to enable averaging across repetitions and participants.

As the intensity and F0 contours are correlated (Whalen and Xu, 1992), and because the intensity contour might be more salient for children with CIs, it might be that pediatric CI users adjust intensity during production to emphasize or deemphasize specific parts of tones. Accordingly, we examined the dynamics of the produced intensity contours. The two left panels of Figure 3 (referring to the non-contrastive syllable) bore a striking similarity with (1) a peak arising about one third of the total duration of the first syllable, and (2) a peak of similar magnitude whether this syllable preceded Tone 1 or Tone 4. For the second syllable, the intensity pattern of Tone 4 closely resembled an inverse V-shape, whereas a high-level intensity was maintained over a longer portion of Tone 1, dropping much closer to the edge of the time window (like an inverse U-shape). It was also apparent that, on average, NH children strengthened the intensity of the second syllable relative to the first, for both tones. In contrast, CI children did so for Tone 4 only.

**FIGURE 4:**
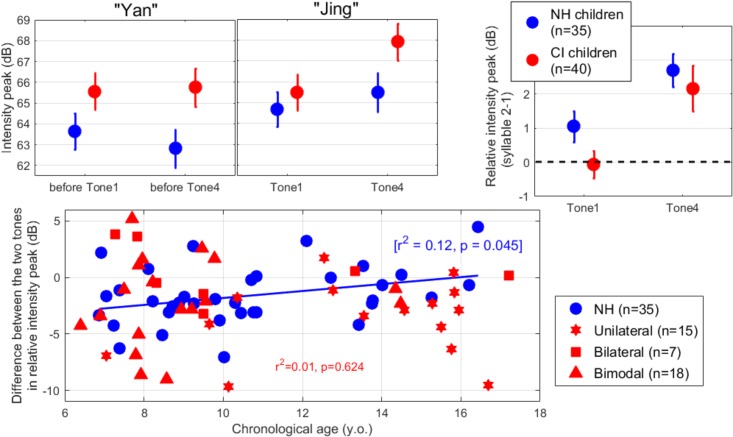
(Top-left) Intensity peak of the first and second syllable of the word “yan-jing” spoken as Tone 1 or Tone 4, averaged across all participants in each population. (Top-right) Intensity peak of each tone relative to the syllable preceding it. All children tended to soften the intensity of Tone 1 relative to Tone 4. (Bottom) Difference in relative intensity peak between the two lexical tones, plotted across participants as a function of their chronological age. A lower negative value is indicative of a stronger tendency to soften Tone 1 relative to Tone 4.

Visual inspection of intensity peak of each syllable (top-left panels of Figure 4) indicates that NH children produced the second syllable at a higher intensity than the first, in both target words (i.e., *eyes* and *eyeglasses*). Children with CIs, on the other hand, did not when producing the target word with Tone 1 (i.e., *eyes*). The LME analysis was performed on the intensity peak of the second syllable, relative to the peak of the first syllable (top-right panel of Figure 4). There was no effect of hearing status [*t*(446) = -1.9, *p* = 0.061], an effect of contrast [*t*(446) = 3.1, *p* = 0.002], and no interaction [*t*(446) = 0.8, *p* = 0.411].

We further examined the individual differences in contrasting the two lexical tones, in relative intensity peak across participants (bottom panel). Here, a negative value indicates that the participant produced a softer intensity for Tone 1 than for Tone 4 (intensity being normalized by what occurred in the first syllable). This was the case for 77% of the participants with NH and 73% of the participants with CIs. The difference was found to be weakly related to chronological age only among NH children (*p* = 0.045, although this would not survive Bonferroni correction). Among children with CI, this behavior was not predicted either by age at implantation (*p* = 0.638) or length of device experience (*p* = 0.833). In summary, all children utilized intensity to some degree to contrast the two tones.

### F0 Pattern

**FIGURE 5:**
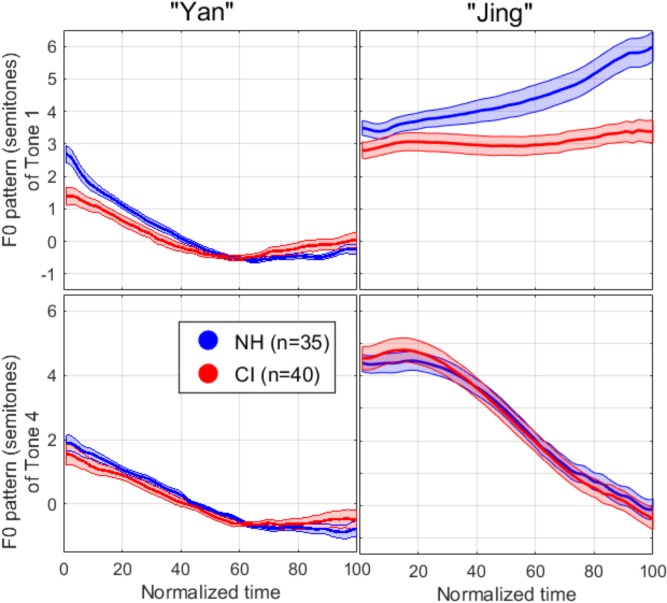
F0 pattern extracted for each syllable in each tone, with normalized duration (extracted with a 20-dB cutoff from the intensity peak) and normalized in its scale by expressing F0 in semitones relative to the mean over the first syllable. Lines represent the mean over all participants in a given group and areas represent one standard error of the mean.

Figure 5 shows the mean F0 pattern for each syllable in each lexical tone, normalized in duration (by resampling 100 points over the length of the pattern) and normalized in its scale (by expressing F0 in semitones relative to the mean over the first syllable). The F0 contour exhibited in the first syllable (left panels) was supposedly a falling-rising contour, but this pattern was washed away to some degree in the averaging process, since the timing of the local minimum varied considerably across repetitions and across participants. More importantly, this pattern was similar whether it preceded Tone 1 or Tone 4, decreasing within a 2–3 semitones scale, and similar for both subject groups, allowing for a consistent reference with which to compare F0 patterns in the second syllable. The top-right panel of Figure 5 shows that participants with NH expressed Tone 1 by starting about 3 semitones above the preceding syllable and slowly raised their voice pitch to another 2–3 semitones higher. Participants with CIs also started about three semitones above the preceding syllable but did not raise their voice over the course of the tone. Both participant groups expressed Tone 4 by dropping their voice pitch by 4–5 semitones (bottom-right panel). Two specific analyses were performed, one based on the F0 median relative to the precedent syllable, and the other based on the F0 movement calculated as the difference between the first and last 30-ms portion.

### F0 Median

**FIGURE 6:**
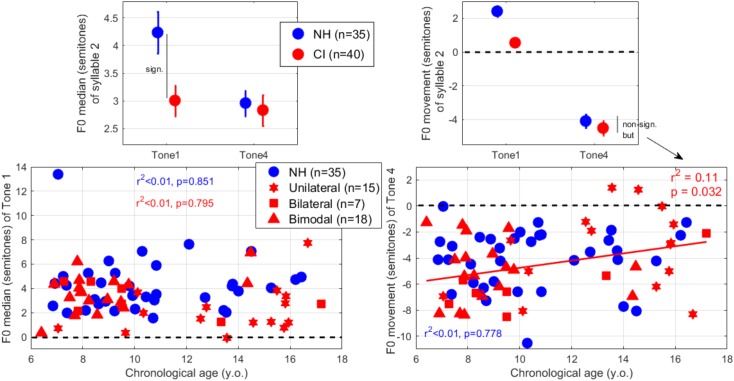
(Top-left) F0 median over the second syllable, expressed relatively to that in the first syllable. A higher value implies the use of a higher pitch range relative to syllable 1, and is particularly relevant for Tone 1’s examination. (Top-right) F0 movement calculated as the difference between the last and first 30-ms of the F0 pattern over the second syllable only. A negative value means a falling inflection, and is particularly relevant for Tone 4’s examination. (Bottom-left) F0 median for Tone 1 and (bottom-right) F0 movement for Tone 4, plotted across participants as a function of their chronological age.

The LME analysis revealed an effect of hearing status [*t*(446) = -2.7, *p* = 0.007], an effect of lexical tone contrast [*t*(446) = -4.1, *p* < 0.001], and an interaction [*t*(446) = 2.6, *p* = 0.009]. As displayed in the top-left panel of Figure 6, participants with NH raised their voice pitch relative to the first syllable by over 4 semitones to express Tone 1, whereas participants with CIs did it to a smaller degree. Seen across participants (bottom-left panel), twelve children with CI changed their voice pitch between the syllables by fewer than two semitones, whereas practically all NH children did it by more than two semitones. This is evidence that at least a fraction of the CI population exhibited a relatively monotonous production since they were not able to indicate Tone 1 as “high” (although they were able to indicate it as “flat” – see next section).

### F0 Movement

The LME analysis revealed an effect of hearing status [*t*(446) = -4.7, *p* < 0.001], an effect of contrast [*t*(446) = -13.8, *p* < 0.001], and a significant interaction [*t*(446) = 2.2, *p* = 0.027]. As displayed in the top-right panel of Figure 6, NH and CI groups differed primarily in the rising versus flat contour of Tone 1. To produce Tone 1 (not shown), 72% of CI participants exhibited a slightly rising F0 contour while the rest exhibited a downward movement. NH participants produced a more accentuated rising contour which contributed to the difference in F0 median aforementioned. To produce Tone 4 (bottom-right panel of Figure 6), all but two participants exhibited a downward movement (-4.5 semitones on average). Interestingly, younger children were more likely to produce a steep downward movement than were older children.

**FIGURE 7:**
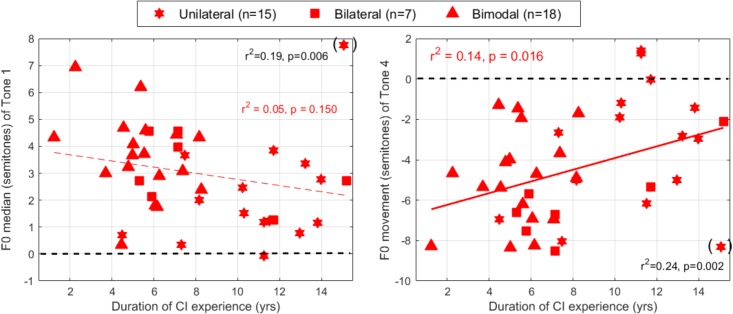
F0 median for Tone 1 (left) and F0 movement for Tone 4 (right) plotted across participants as a function of their years of CI use. The CI participants who had used their CI for the longest time exhibited less modulation of their vocal chords either to differentiate the pitch range between syllables (as in the case of Tone 1) or to indicate the direction of a pitch sweep (as in the case of Tone 4).

We also examined the extent to which these F0 parameters could depend on years of CI use (Figure 7). This experience-related factor was a stronger predictor than chronological age in explaining how much participants with CIs dropped their voice pitch within Tone 4. As displayed on the right panel, participants with the longest experience with their CIs produced Tone 4 with the shallowest falling slope (*p* = 0.016) accounting for 14% of the variance. Note that excluding one subject with 15 years of experience (16.7 years old, the second oldest of our sample) whose productions were very good, this relationship strengthened considerably (*r*^2^= 0.24, *p* = 0.002). In addition, there was a non-significant trend, where the long-term users produced smaller differences in F0 median between the two syllables when producing Tone 1 (left panel), and this relationship was considerably strengthened by ignoring the same 16.7 years old subject (*r*^2^= 0.19, *p* = 0.006). Despite a large inter-subject variability that is often observed among CI users, there is some evidence that long-term CI experience was associated with a more monotonous F0 production.

### Role of Acoustic Hearing

Although all participants were tested with their earlier-implanted CI only, they varied in their everyday device configurations. A between-subjects analysis of variance in the production outcomes discussed above (1: difference between the two tones in duration ratio, 2: difference between the two tones in intensity ratio, 3: F0 median over Tone 1 relative to the preceding syllable, and 4: F0 movement over Tone 4) was conducted one by one, with Bonferroni corrections, based on whether the listeners were Bimodal (*N* = 18, 45%), Unilateral-CI (*N* = 15, 37.5%) or Bilateral-CI (*N* = 7, 17.5%) users. The results did not show consistent patterns. No significant differences were observed between the groups in duration characteristics [*F*(2,37) = 2.4, *p* = 0.108], and only a marginal difference in intensity characteristics [*F*(2,37) = 3.3, *p* = 0.046] driven by a significant difference between Unilateral-CI and Bilateral-CI users (*p* = 0.036). Another marginal effect of group was observed for F0 median [*F*(2,37) = 3.1, *p* = 0.059], driven by a difference between Unilateral-CI and Bimodal listeners, with Bimodal listeners producing a higher F0 median than Unimodal listeners for Tone 1 (*p* = 0.047). However, this effect was unlikely to be due to residual hearing, because there was no difference between Bilateral-CI and Bimodal users (*p* = 0.780). Finally, differences between the groups in F0 movement also failed to reach significance [*F*(2,37) = 3.0, *p* = 0.064], and did not point either toward a benefit of residual hearing (i.e., Bimodal users producing F0 drops of about -5 semitones, while Bilateral-CI and Unilateral-CI users produced drops of -6 and -3.5 semitones, respectively, and no pairwise comparison reached significance). Also, the groups were different in chronological age, with Unilateral-CI users being significantly older than bimodal users (mean ages 13.4 vs. 8.9 years of age, *p* < 0.001) and marginally older than bilateral users (13.4 vs. 10.4, *p* = 0.055). As duration of device experience co-varied with chronological age, this may have also contributed to the differences between groups.

## Perception Results

**FIGURE 8:**
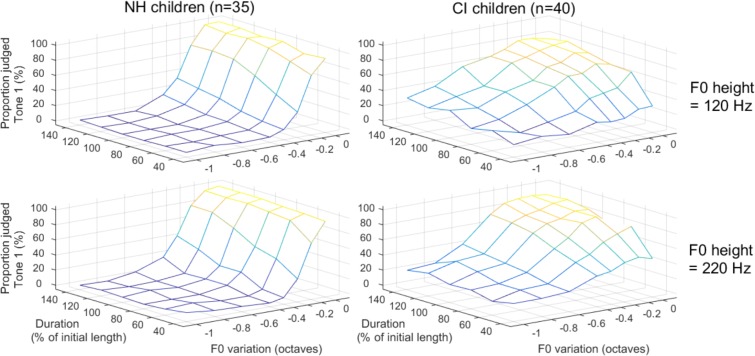
Proportion of stimuli perceived as Tone 1 among a continuum of stimuli varying orthogonally in F0-variation, duration, and F0-height. A steeper slope of the psychometric function along a given dimension (e.g., F0 variation) reflects a stronger reliance on this cue.

**FIGURE 9:**
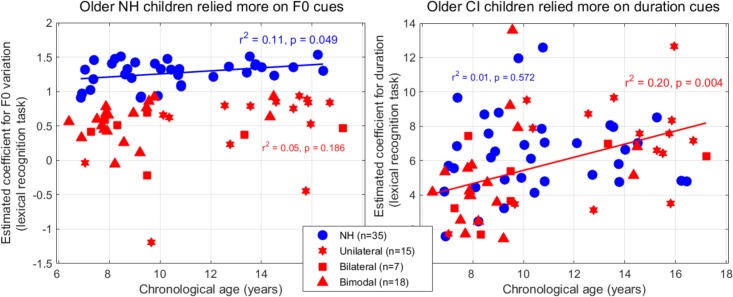
Coefficients extracted from the logistic mixed-effect model reflecting the reliance on F0-variation (left) and duration (right) in the lexical tone recognition task, as a function of the children’s age.

The data for the perception task are shown in Figure 8. A logistic mixed-effect analysis revealed a significant interaction between population and the slope of F0 variation [*t*(20451) = 11.4, *p* < 0.001]. The proportion of Tone 1 responses for NH children rose dramatically from about 20 to 90% (on average over the two F0 heights) as the F0 drop changed in a subtle manner between -0.3 to -0.1 octave. For participants with CIs, the proportion of Tone 1 responses varied more gradually between 20 and 75% over the entire scale of F0 variation. As a consequence, the estimated coefficient for F0 variation differed between the groups: -20.7 and -4.3 for NH and CI participants, respectively (Figure 9). There was a main effect of duration [*t*(20451) = 10.4, *p* < 0.001], favoring Tone 1 responses the longer the syllable. Its estimated coefficient was 6.6, and it did not differ between NH and CI participants [*t*(20451) = -0.5, *p* = 0.604]. Finally, there was an interaction between the two experimental manipulations, F0 variation and duration [*t*(20451) = -4.9, *p* < 0.001], which itself interacted in a 3-way with population [*t*(20451) = 2.8, *p* = 0.005]. This can be appreciated when considering that NH children made use of duration only when the pitch contours were ambiguous (F0-slopes of -0.3 to -0.1 octave), whereas CI children made use of duration cues throughout all manipulations. Notably, at the extremes (for CI children): extending the duration from 40 to 140% still caused a +10% increase in Tone 1 responses when the F0 contour dropped by a full octave, and caused a +45% increase in Tone 1 responses when the F0 contour was flat.

Note that F0-height was not included as a fixed factor, as it did not represent an experimental manipulation but was included to represent the natural variability in lexical tones (male or female voices). It made overall little difference to the responses (top versus bottom panels, Figure 8), and estimates of the per-subject random slopes allocated to F0-height did not differ between participants with NH and with CIs [*t*(73) = -1.3, *p* = 0.207]. Also, F0-height did not correlate with any of the production parameters, and is not discussed any further.

Estimates of the per-subject slopes for F0-variation and duration were plotted across participants (Figure 9). To homogenize the variability between the two populations, the estimates for F0-variation were expressed in log_10_ of the absolute value. There was an age effect among participants with NH (*p* = 0.049, although it would not survive Bonferroni correction), whereby the older children placed slightly more weight (than the younger children) on the slope of F0 variation. In contrast, there was an age effect among participants with CIs, whereby the older children placed more weight on duration, explaining 20% of the variance (*p* = 0.004). Note that the present participants in the CI group were implanted before age 3 (median = 2.5 years of age); their chronological ages correlated strongly with the length of CI experience [*r*^2^ = 0.72, *p* < 0.001], and a similar correlation could therefore, be obtained when the variable *chronological age* was substituted with the *length of CI experience* [*r*^2^ = 0.19, *p* = 0.005]. Additionally, the participants with NH did not exhibit the same trend when compared to that of the participants with a CI [*r*^2^ = 0.01, *p* = 0.572]. Taken together, these findings suggest that chronological development itself does not contribute to the observed trend that older participants with a CI placed more weight on duration cues compared to those younger ones. In other words, rather than a developmental factor, this effect could well be driven by the opportunity to have learned cue-trading through the experience with CIs.

**FIGURE 10:**
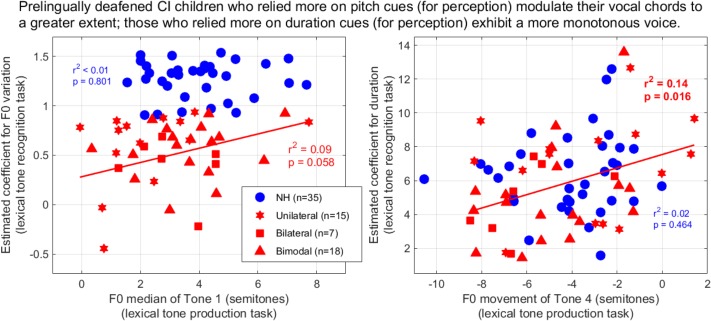
Coefficients extracted from the logistic mixed-effect model reflecting the reliance on F0-variation (left) and duration (right) in the lexical tone recognition task, as a function of the F0 parameters extracted from production. Coefficients for F0 variation are expressed in log_10_ of their absolute value to improve homogeneity of variance between the two participant groups.

Finally, we addressed the question of whether the perceptual weights that a given child placed upon F0 contours and duration cues could be related to the production outcomes discussed earlier. For the NH group, we did not observe any relationship. For the CI group, however, two interesting correlations were observed. First, the participants who placed greater weight to duration cues perceptually were the individuals who exhibited little downward movement when producing Tone 4 (*p* = 0.016, right panel of Figure 10). This is exemplified by the two participants who relied the most on duration (coefficient of 13–14), and despite being quite different in age (9.5 vs. 16), they both expressed Tone 4 with less than a two semitones drop. Second, there was a marginal correlation (*p* = 0.058, left panel), where the users who relied more on F0 perceptually tended to raise their voice pitch more between Tone 1 and the syllable preceding it. An account based on the sensitivity to F0 would easily explain such a link: the users who are lucky enough to discriminate a static F0 difference of 1–2 semitones (Deroche et al., 2014) or track a F0 glide down to 8 semitones/s (Deroche et al., 2019) in the voice of other speakers could afford to rely on F0 contours perceptually even though the trajectory of the F0 inflection is coarse, and similarly this sensitivity may be just enough for their auditory feedback to exhibit this coarse inflection in their own production. Therefore, it could prevent the “monotonizing” impact of hearing through a CI over many years (Figure 7). However, it must be acknowledged that the perceptual weights on duration did not correlate with the production outcome respective to duration ratio (*p* = 0.734); and the perceptual weights on F0 variation did not correlate with the F0 movement of Tone 4 (*p* = 0.266). Therefore, on the whole, our hypothesis that “reliance on specific acoustic dimensions in the identification of tones would be reflected in their production by the same individuals” received mixed support.

## General Discussion

Peng et al. (2017) reported that pediatric CI recipients used both F0 and duration cues to discriminate between Tones 1 and 4, while NH peers relied exclusively on F0 cues. This result seemed consistent with cue-trading, in which CI listeners use alternative acoustic dimensions that co-vary with F0 contour to compensate for the limited functional spectral resolution. However, when the same children were asked to identify lexical tones in a set of 40 naturally spoken words, their performance was predicted by their reliance on F0 rather than on duration cues. This makes sense considering that, in connected speech, the four lexical tones actually show little difference in duration. With minimal semantic or linguistic context, it is hard to see how those children could indeed make use of duration cues. In other words, while pediatric CI users may rely on amplitude and/or duration cues as additional sources of information to perceive lexical tones, it is their sensitivity to F0 contours that predicts lexical tone recognition in everyday listening. Duration cues may not be very helpful at the sentence level, and as such, the degraded F0 contour may still be the more reliable information in ecological situations.

This begged the question of whether CI children would still attempt to modulate their voice pitch despite ignoring how well they succeeded in doing so, or whether they would attempt to convey those tones via other dimensions that they have adequate representation of, even though these co-varying cues may not be relied upon by NH listeners. The present results provided several key points, as follows.

First, pediatric CI recipients produced Tone 4 shorter than Tone 1 (Figure 2). This behavior simply exaggerated that of NH participants (more easily observable with voicing duration), which is why CI users were able to produce meaningful tone distinctions using duration cues. This finding highlights that the patterns produced by all participants reflect to some degree the biological or mechanical constraints of human vocal production. Thus, CI users cannot produce tones in an arbitrary way; they can only refine their productions based on what is acceptable and meaningful in the natural lexicon. On a side note, it is notable that the participants with CIs exhibited longer vowels than their NH peers, and this may not be coincidental. By slowing down their speech overall, these children increase their capacity to shorten specific syllables when they need it, without reaching a narrow window, where this might conflict with audibility. Additionally, speaking slowly tempers fast fluctuations in intensity, i.e., it gives them a better control over loudness changes. These results are consistent with findings of Chuang et al. (2012), where CI children are reported to exhibit longer vowels as well as longer pauses between words, resulting in a slower speaking rate than their NH peers matched in age, sex, and educational level.

Second, while individuals with NH stressed the second syllable relative to the first in both Tones (relying on pitch to convey the tone identity), pediatric CI recipients tended to soften Tone 1 relative to the syllable preceding it (Figure 4). However, we did not capture a trait of the intensity pattern that would highlight a significant interaction between population and tone. Rather, a marginal effect of group (*p* = 0.061) showed that participants with CIs simply did not emphasize the second syllable as much as their NH peers did. Perhaps, they are less aware of which syllable contains to the critical information that distinguishes the two tones and consequently do not feel the need to emphasize it. Arguably, compression of dynamic range in the auditory feedback that CI children received from their own voice should hinder their ability to detect small increments in loudness. However, this explanation suffers from the clear difference between the two tones (>2 dB) that CI children successfully exhibited (as NH children did). Another factor that could have played a role here is the fact that NH children listened binaurally to their voice’s feedback while CI users listened monaurally (regardless of whether they used another CI or a HA in their everyday life). This means that several of the CI children did not experience the binaural summation they are normally used to experience, and this could have led them to speak louder (for both tones).

Third, there were signs (although subtle) of atypical F0 productions among some pediatric CI recipients. This was reflected by a lower tendency to (1) mark the F0 median of Tone 1 as *higher* than the syllable preceding it, and (2) mark the F0 movement of Tone 4 as *falling*. However, several notes of caution must be acknowledged. The first observation was partly accounted for by a difference in F0 movement between the two groups. Children with NH actually raised the F0 over the course of Tone 1 by 2 semitones, while children with CI produced it as flat (as it is supposed to be, in isolation). This raises a doubt about NH children’s production quality which must be answered. Much of the literature on Mandarin’s phonetics is based on monosyllabic words. Xu (1997) demonstrated that with bi-tonal sequences, there are anticipatory and carry-over effects to consider, and most relevant here, “*a considerable portion of the F0 curve for Tone 1 has a rising contour when the preceding tone is Tone 3 or Tone 4, both of which have a low offset*” (p. 69). Thus, the present behavior of NH children is perfectly normal and expected, given that Tone 3 was used in the first syllable. In contrast, the fact that CI children stuck to the canonical form of Tone 1 indicates that they did not take the tonal context into consideration. The second observation is also debatable since, at a population level, there was no difference between the two groups in F0 movement for Tone 4; only the older CI users reduced the degree of their drop in F0. Therefore, in both measures (F0 median for Tone 1 or F0 movement for Tone 4), we think that the interesting finding is about the effect of CI experience (Figure 7), rather than a deficit of the entire population.

The effect of CI experience raises discrepancies among earlier studies. In earlier studies, the quality of lexical tone productions was rated (by a NH adult or a machine), and those ratings were dominated by the quality of the F0 contour. Hence, such ratings should in principle be consistent with acoustic parameters such as those presented here. Han et al. (2007) reported that CI experience was *beneficial* to production ratings (with *N* = 14). However, Zhou et al. (2013) failed to replicate this finding with a large sample size (*N* = 110). Earlier, Peng et al. (2004) did not find such a benefit of experience (with *N* = 30) and this was not possible for Xu et al. (2004) to investigate, as their sample included only 4 CI children. Here, we found CI experience to be rather *detrimental* to the quality of F0 productions. Arguably, the linear trends were modest (e.g., accounting for only 14% of the variance in the right panel of Figure 7), but we note that those CI users who produced the shallower falling tone were also the subjects who placed greater perceptual weights on duration (Figure 10, right panel, also accounting for 14% of the variance). We do not believe this relation to be coincidental, and it suggests a “monotonizing” process (and a shift in perceptual strategies) that takes place over years of hearing through the device, perhaps as many as 20 years given the current slopes. The most trivial interpretation is that the poor feedback of voice pitch provided by current CIs reinforces the percept of a monotonous voice and over many years, some CI users adapt and no longer modulate their vocal chords (as this seems to have no impact on their auditory feedback). This being said, children with CIs have ample opportunities to receive direct or indirect feedback from caregivers, teachers, clinicians, and other NH children, on how to produce better F0 contours to enhance their intelligibility. These interactions should mitigate the saliency of the monotonous voice percept, but perhaps it is difficult to learn F0 control from outsiders’ advice.

Rather than a “monotonizing” impact of CI experience, an alternative explanation is that pediatric CI recipients exhibited a stronger developmental trajectory in their F0 control than did their NH peers. Adults and older children generally speak with narrower fluctuations in F0 than do young children (e.g., review by Kent, 1976). Older CI users could have spoken on a narrow range of F0 fluctuations, not because their voice was monotonous per se, but because they had already refined the control of their vocal chords to operate within a range that is just enough to convey the tonal information. This interpretation suffers from two weaknesses: (1) the correlations with CI experience were stronger than those with chronological age of CI children (Figure 7 vs. Figure 6), and (2) there was no effect of chronological age among NH children (Figure 6). However, the developmental trajectories of CI children are known to differ from those of NH children, and it is easy to imagine that the refinement process in F0 variability requires hearing. So, this interpretation should certainly not be discarded until one can test precisely whether these F0 fluctuations would eventually (with very long-term exposure) flatten or show a similarly narrow distribution as for NH adults.

One of the most efficient ways to disambiguate such interpretations is to examine production with and without auditory feedback, i.e., by turning the CI on and off. Such studies differ in their outcome, with some showing differences between the two conditions (Poissant et al., 2006; Bharadwaj et al., 2007) and others finding no difference in the acoustics of their speech (Tye-Murray et al., 1996; Turgeon et al., 2017). Applied to lexical tones, similar designs would be greatly informative. Also critical is the fact that the mechanics of speech production may actually differ (e.g., when the feedback is on or off) even when no acoustic difference is observed in the recordings, which is why articulatory measures may eventually be necessary to fully understand the abnormal vocal production by CI users and their relation to experience-related plasticity (Turgeon et al., 2015, 2017).

Fourth, a number of results in the lexical tone recognition task were found to be consistent with previous findings (Peng et al., 2017): (1) the tonal boundary along a continuum of F0 slopes was very sharp for NH children but more gradual for CI children; (2) the tonal boundary along a continuum of compressed to stretched syllables was generally shallower (than for the F0 slope) and CI children relied on duration across the entire range of F0 slopes, whereas NH children used it only in very few cases, where the F0 slope was ambiguous; (3) as they got older, NH children relied even more on F0 cues while CI children relied even more on duration cues. This latter finding is particularly important because it is potentially the reason why prelingually deafened CI users improve over time in this task, i.e., not because they somehow get better at processing F0 contours but because they have learnt to detect other cues. This interpretation would seem consistent with a study by Tao et al. (2015) who observed considerable deficits in melodic contour identification by Mandarin-speaking CI users (aged 6–26 years) while performing well in a lexical tone recognition task. Also, performance in the two tasks was not correlated among the 33 users in their study (children and adults, pre- or post-lingual). The authors concluded that CI users must compensate their deficits in F0 processing by using the amplitude and duration cues in lexical tones. Note that this learning to trade among cues must take place while hearing, but among prelingually deafened children, it is always difficult to disentangle developmental effect from that of CI experience itself. Zhou et al. (2013) reported CI experience to account for 18% of the variance in lexical tone identification; in very good agreement, we found it to account for 19%.

Finally, the present study focused on the contrast between Tone 1 vs. Tone 4, as this pair permitted us to examine the changes in perceptual weighting between two acoustic dimensions (F0 and duration) known to contribute to lexical tone recognition for Mandarin Chinese. This Tone 1 vs. Tone 4 contrast is also suitable for our targeted patient populations and listeners who are relatively young in age, given the relatively simple linguistic meanings of the chosen bi-syllabic words with these two lexical tones (i.e., *eyes* vs. *eyeglasses*), in addition to the fact that they do not involve complex contour changes as with Tones 2 and 3 (Peng et al., 2017). Ideally, it would be necessary to replicate the present findings with other pairs of lexical tones and considering different tonal context environments.

## Conclusion

This study analyzed acoustic recordings of Mandarin Chinese, pediatric CI recipients, and their age-matched peers with NH. All participants were asked to produce disyllabic words with contrastive lexical tones (i.e., Tones 1 and 4). Pediatric CI recipients, at least the older and more experienced ones, exhibited narrower modulations of their voice pitch (both within and across syllables). However, it remains unclear whether this represents a “monotonizing” impact of CI experience or rather a refinement in the control of vocal chords to convey the tonal information more like adults. Perhaps as a compensatory mechanism, CI children contrasted the duration properties of the second syllable that distinguish Tones 1 and 4. To explore this interplay further and link it to perception, the same children took part in a lexical tone recognition task, discriminating among parametric variations of many tokens in a Tone 1–Tone 4 continuum. The perceptual weights extracted from this task confirmed that CI children relied less on F0 cues than did NH children. CI children used duration cues all the time, whereas NH children used them only when F0 cues were ambiguous. CI children who placed greater weight on duration cues also tended to have the most monotonous tone production. This result supports the idea that perception and production are reasonably coupled, even with this clinical population having an auditory feedback of relatively poor quality.

## Ethics Statement

This study was carried out in accordance with the recommendations of the Institutional Review Board at the Chi-Mei Medical Center which approved the protocol. All subjects gave written informed consent in accordance with the Declaration of Helsinki.

## Author Contributions

MD analyzed the data and wrote the manuscript. H-PL collected all the data. Y-SL was responsible for the project supervision in Taiwan. MC obtained funding for this research program, worked closely with S-CP on the rationale, with MD on the analyses, and edited the manuscript. S-CP developed the rationale, designed the experimental tasks, contributed to analyses, and edited the manuscript.

## Conflict of Interest Statement

The authors declare that the research was conducted in the absence of any commercial or financial relationships that could be construed as a potential conflict of interest.

## Appendix

Throughout visual screening of each production (see examples in Figure 1), we noted that the second syllable often exhibited an elevation of F0 from the last phoneme, especially for Tone 4 (e.g., right panels in Figure 1). This is the kind of observation that led us to reiterate the analysis with a 10-dB cutoff to selectively capture the stereotypical shape of the tones, even though it necessarily restricted the amplitude of F0 movements. Here, we report on the statistical results derived for duration, F0 median, and F0 movement with the narrower window.

The duration of syllables extracted with a 10-dB cutoff provided a closer estimation of the voicing duration. On average, this voicing duration was reduced by about 100 ms compared to the syllable duration (which was described in Figure 2, top-left panels), but the overall pattern was largely similar. When expressed as a ratio between the two syllables, the LME analysis revealed no effect of hearing status [*t*(446) = 1.1, *p* = 0.259], an effect of contrast [*t*(446) = -5.0, *p* < 0.001], and an interaction between the two [*t*(446) = -2.2, *p* = 0.028]. Those results were therefore, qualitatively similar as those revealed with the 20-dB window, but with a stronger role for contrast (tone 4 being overall less voiced than tone 1).

When expressing F0 median in semitones relative to the first syllable, the LME analysis revealed a marginal effect of hearing status [*t*(446) = -2.0, *p* = 0.045], a marginal effect of contrast [*t*(446) = -1.8, *p* = 0.069], and a critical interaction between the two [*t*(446) = 2.4, *p* = 0.015]. When examining F0 movement, the LME analysis revealed an effect of hearing status [*t*(446) = -4.2, *p* < 0.001], a strong effect of contrast [*t*(446) = -14.9, *p* < 0.001], and a critical interaction between the two [*t*(446) = 2.3, *p* = 0.022]. Again, these outcomes were similar to the ones aforementioned. Overall, therefore, the size of the window considered (20 or 10 dB drop from the intensity peak) made qualitatively no difference to the results described in the rest of the manuscript.
